# Supplementary material for: Plantar threshold sensitivity assessment using an automated tool—Clinical assessment comparison between a control population without type 2 diabetes mellitus, and populations with type 2 diabetes mellitus, with and without neuropathy symptoms
Source: PLoS One. 2023 Jul 7;18(7):e0286559. doi: 10.1371/journal.pone.0286559 (PMC10328367; doi:10.1371/journal.pone.0286559)
Supplement: S1 Dataset — (PDF) [file pone.0286559.s001.pdf]

Group 1 Subjects							
Patient ID	Sex	Age (years)	BMI (kg/m^2)	ABI (mmHg)	FBS (mg/dL)	HbA1c (%)	TSI Norm
C1	Female	74	26.4	1.36	101.6667	5.643333	13.02681
C2	Male	61	27.4	1.23	102.3333		10.72981
C3	Male	59	41.6	1.3	103		15.55249
C4	Female	59	33.4	1.05	94.66667		8.038518
C5	Female	70	33.3	1.5	95	5.333333	14.79399
C6	Female	67	23.6	1.07	91.33333		15.36504
C7	Male	63	19.7	1.16	95		14.04279
C8	Male	63	38.2	1.16	97		12.64349
C9	Female	46	23	1.02	86.66667	5.256667	7.11493
C10	Male	72	22.2	1	107		17.48371
C11	Male	65	24.2	1.18	103.3333		11.5075
C12	Male	81	22.3	1.02	111	5.9	12.93836
C13	Male	65	33	1.01	97.66667		9.753646
C14	Male	47	28	1.05	87.66667		12.64823
C15	Male	65	30.1	1.17	108.6667	6.196667	16.11707
C16	Female	69	39.6	1.23	103.3333		11.6838
C17	Female	41	22.2	1.24	85.33333	5.04	7.200328
C18	Female	56	22.9	1.05	74.66667		5.61449
C19	Male	74	22.4	1.08	84		10.09325
C20	Male	45	48.6	1.17	115	5.7	7.787026
C21	Male	56	26.8	1.13	80		12.03518
C22	Male	46	20.5	1.3			8.418135

Group 2 Subjects							
Patient ID	Sex	Age (years)	BMI (kg/m^2)	ABI (mmHg)	FBS (mg/dL)	HbA1c (%)	Total Norm
S1	Male	62	33	1.03	203.3333	11.13333	14.20282
S2	Female	66	26.9	1	108.3333	6.013333	15.86821
S3	Male	50	40.2	1.09	158	8.546667	19.59592
S4	Male	42	31.8	1.02	139.6667	6.006667	13.77211
S5	Male	65	28.8	1.03	166	8.796667	12.01629
S6	Male	55	33	1.04	261.3333	11.4	8.679222
S7	Male	54	36.8	1.2	121.6667	6.55	15.2754
S8	Female	67	50.2	1	112.3333	6.366667	13.66163
S9	Male	55	39.9	1.08	119.6667	7.206667	17.67937
S10	Male	76	29.1	1.2	100.6667	6.096667	10.34752
S11	Male	70	34	1.25	198	8.993333	17.41238
S12	Female	67	23.3	1.5	86	5.393333	9.068382
S13	Male	74	32.3	1.05	136	6.166667	10.37413
S14	Female	64	39.9	1.33	124	8.293333	9.219198
S15	Male	79	22	1.45	114.3333	6.283333	15.37057
S16	Male	55	22.6	1.07	126.6667	6.29	8.844835
S17	Female	57	36.8	1.09	239	7.963333	15.65815
S18	Female	68	39	1.05	192	8.533333	12.5774
S19	Female	79	26.8	1.21	143	6.5	12.98208
S20	Female	58	44.3	1	158.3333	8.366667	16.49432
S21	Male	78	27	1.4	167.6667	7.65	9.500234
S22	Female	65	30.6	1.32	168.3333	6.85	8.397949
S23	Male	62	26.7	1.36	142.6667	7.186667	7.604933
S24	Female	64	26.5	1.04	84.33333	5.346667	16.72098
S25	Male	84	30.7	1.25	138.3333	7.82	19.59592
S26	Female	73	34.4	1	142	8.07	16.12335

Group 3 Subjects							
Patient ID	Sex	Age (years)	BMI (kg/m^2)	ABI (mmHg)	FBS (mg/dL)	HbA1c (%)	Total Norm
NS1	Female	68	22.7	1.18	103.6667	5.29	11.21467
NS2	Female	58	31.3	1	105.6667	6.21	4.734742
NS3	Male	58	32.9	1.08	150	7.843333	16.73679
NS4	Female	58	33.5	1.03	141.3333	7.09	3.279566
NS5	Female	69	30.2	1.19	157.6667	7.263333	11.11775
NS6	Male	64	30.4	1.09	110.6667	5.873333	13.03244
NS7	Male	63	35.2	1.2	161	7.35	17.10672
NS8	Male	64	34.9	1.3	157.3333	7.866667	3.703452
NS9	Female	61	34.4	1.26	128.3333	6.263333	12.15529
NS10	Male	63	27.3	1.19	129.3333	6.296667	10.93882
NS11	Female	64	37.9	1.06	159	7.75	5.303248
NS12	Female	63	43.7	1	126	6.76	16.96231
NS13	Male	64	35.67	1.41	170	6.796667	17.54993
NS14	Female	76	36.7	1.1	132	6.42	3.072458
NS15	Female	74	43.3	1.01	114.6667	5.693333	9.091266
NS16	Female	74	30.6	1.07	133.3333	6.31	5.318965
NS17	Male	66	24.2	1.18	139	7.65	16.70359
NS18	Male	72	36.4	1.05	160.3333	8.173333	18.66976
NS19	Male	60	33.4	1.12	129.6667	6.74	8.936271
NS20	Female	67	23.4	1.03	144	6.373333	5.73062
NS21	Female	65	22.1	1.06	108.3333	6.59	6.657327
NS22	Female	78	27	1.03	119	6.396667	10.34988

Group 1 Subjects- Threshold Sensitivity per Location

Patient Identifier	Right Toe 1	Right Toe 2	Right Toe 3	Right Toe 4	Right Toe 5	Left Toe 1	Left Toe 2	Left Toe 3	Left Toe 4	Left Toe 5	Right Ball 1	Right Ball 2	Right Ball 3	Right Ball 4	Right Ball 5	Left Ball 1	Left Ball 2	Left Ball 3	Left Ball 4	Left Ball 5	Right Heel 1	Right Heel 2	Right Heel 3	Left Heel 1	Left Heel 2	Left Heel 3
C1	0.35	0.35	8	0.35	0.7	0.7	>10.0	10	>10.0	0.35	8	>10.0	0.7	10	0.35	8	6	6	>10.0	0.35	>10.0	8	>10.0	>10.0	0.7	>10.0
C2	0.35	>10.0	0.35	>10.0	0.7	0.35	0.35	0.35	2	2	10	0.35	0.35	2	0.7	0.35	0.7	>10.0	>10.0	>10.0	8	10	8	>10.0	0.35	4
C3	4	0.35	>10.0	2	0.35	8	2	0.35	0.7	6	>10.0	>10.0	>10.0	>10.0	>10.0	>10.0	>10.0	8	>10.0	10	0.7	>10.0	>10.0	>10.0	>10.0	>10.0
C4	0.35	0.35	0.35	>10.0	0.35	0.35	0.35	0.35	0.35	0.35	0.35	0.35	0.35	0.7	0.35	0.35	8	>10.0	0.35	6	0.7	0.35	0.7	>10.0	0.7	>10.0
C5	>10.0	4	4	>10.0	>10.0	0.35	10	0.35	6	0.35	>10.0	>10.0	10	>10.0	>10.0	>10.0	>10.0	10	>10.0	>10.0	6	4	10	0.7	4	10
C6	>10.0	10	>10.0	6	10	>10.0	8	>10.0	>10.0	0.35	10	8	>10.0	6	0.35	>10.0	10	2	10	0.35	0.35	>10.0	10	>10.0	>10.0	>10.0
C7	4	0.7	0.35	0.35	2	2	0.35	0.35	>10.0	0.35	0.35	0.35	6	>10.0	2	6	>10.0	>10.0	>10.0	4	>10.0	>10.0	>10.0	>10.0	>10.0	>10.0
C8	0.35	0.35	>10.0	10	>10.0	>10.0	>10.0	2	>10.0	0.7	2	>10.0	10	6	>10.0	0.35	0.35	10	>10.0	>10.0	>10.0	0.35	2	0.35	6	>10.0
C9	10	2	0.35	0.7	0.35	4	0.35	0.35	0.35	0.35	0.35	0.7	0.7	0.7	>10.0	0.35	4	0.35	10	10	0.7	10	0.7	0.35	0.35	0.7
C10	4	>10.0	10	10	10	>10.0	0.35	>10.0	0.35	>10.0	0.7	>10.0	>10.0	>10.0	>10.0	>10.0	>10.0	>10.0	10	>10.0	>10.0	>10.0	>10.0	>10.0	>10.0	>10.0
C11	0.35	>10.0	0.35	0.35	0.7	>10.0	>10.0	2	>10.0	0.35	10	>10.0	0.7	IDE	>10.0	0.35	0.7	>10.0	0.35	4	6	6	>10.0	2	4	0.7
C12	0.35	>10.0	>10.0	>10.0	6	8	>10.0	10	>10.0	>10.0	2	0.35	0.7	0.35	0.7	0.35	>10.0	>10.0	>10.0	0.35	IDE	4	0.35	2	>10.0	>10.0
C13	0.7	0.7	0.35	>10.0	0.35	6	0.35	0.35	>10.0	IDE	0.35	>10.0	0.35	0.35	0.7	2	2	10	>10.0	0.35	0.35	0.35	>10.0	>10.0	>10.0	0.7
C14	0.7	2	>10.0	0.7	0.7	10	0.7	>10.0	0.7	0.7	4	>10.0	8	2	4	IDE	>10.0	10	10	0.7	0.7	>10.0	0.7	8	>10.0	>10.0
C15	>10.0	>10.0	>10.0	0.35	>10.0	0.7	8	2	2	>10.0	10	>10.0	>10.0	>10.0	>10.0	0.35	10	8	>10.0	>10.0	>10.0	0.7	>10.0	>10.0	IDE	>10.0
C16	6	2	0.35	0.7	0.35	0.7	2	0.7	0.35	2	8	8	8	4	0.35	4	10	0.7	>10.0	>10.0	6	0.35	0.7	>10.0	IDE	>10.0
C17	0.35	0.35	0.35	IDE	0.35	0.35	0.35	0.35	0.35	0.35	0.35	0.35	6	IDE	>10.0	0.35	0.35	0.35	10	10	0.35	4	>10.0	2	0.7	0.7
C18	0.35	0.35	0.35	2	0.35	0.35	0.35	0.35	0.35	0.35	0.35	IDE	0.35	10	0.7	0.7	0.7	0.7	0.35	2	0.35	0.35	IDE	0.7	8	4
C19	0.35	0.35	2	0.35	0.35	2	0.35	0.35	2	0.35	>10.0	0.7	0.7	>10.0	IDE	IDE	6	4	0.7	>10.0	10	>10.0	4	6	0.7	0.7
C20	0.7	0.35	0.7	0.35	0.7	0.7	0.35	0.35	0.35	0.35	0.7	10	2	>10.0	>10.0	0.7	6	IDE	10	4	0.35	0.7	IDE	0.7	0.35	0.7
C21	4	>10.0	4	>10.0	IDE	0.35	10	10	>10.0	2	>10.0	>10.0	6	IDE	10	>10.0	0.35	2	IDE	0.7	2	0.35	4	0.35	8	4
C22	6	0.7	0.35	10	0.35	2	0.35	0.35	4	0.35	4	4	6	IDE	0.7	2	0.35	>10.0	IDE	10	0.7	0.7	0.7	0.7	0.7	>10.0

Group 2 Subjects- Threshold Sensitivity per Location

Patient Identifier	Right Toe 1	Right Toe 2	Right Toe 3	Right Toe 4	Right Toe 5	Left Toe 1	Left Toe 2	Left Toe 3	Left Toe 4	Left Toe 5	Right Ball 1	Right Ball 2	Right Ball 3	Right Ball 4	Right Ball 5	Left Ball 1	Left Ball 2	Left Ball 3	Left Ball 4	Left Ball 5	Right Heel 1	Right Heel 2	Right Heel 3	Left Heel 1	Left Heel 2	Left Heel 3
S1	0.35	0.35	0.35	0.35	0.35	>10.0	>10.0	>10.0	>10.0	>10.0	10	0.35	2	0.35	0.35	0.35	>10.0	>10.0	10	>10.0	8	0.35	>10.0	>10.0	>10.0	>10.0
S2	>10.0	4	0.35	>10.0	>10.0	0.35	>10.0	>10.0	10	>10.0	>10.0	>10.0	2	0.7	>10.0	>10.0	6	4	>10.0	>10.0	>10.0	>10.0	>10.0	>10.0	0.7	>10.0
S3	>10.0	>10.0	>10.0	>10.0	>10.0	>10.0	>10.0	>10.0	>10.0	>10.0	>10.0	>10.0	>10.0	>10.0	>10.0	>10.0	>10.0	>10.0	>10.0	>10.0	>10.0	>10.0	>10.0	>10.0	>10.0	>10.0
S4	>10.0	>10.0	0.35	>10.0	>10.0	0.35	0.35	10	0.7	4	0.35	10	>10.0	>10.0	0.35	0.7	0.35	0.35	>10.0	>10.0	10	>10.0	0.7	>10.0	>10.0	>10.0
S5	0.35	0.7	2	10	0.35	>10.0	2	0.35	>10.0	>10.0	6	>10.0	>10.0	10	0.35	>10.0	6	4	10	10	0.35	0.35	0.35	0.35	>10.0	>10.0
S6	4	0.7	0.35	0.7	0.35	0.35	0.35	0.35	0.7	0.35	0.35	0.35	0.7	8	>10.0	8	0.35	0.35	>10.0	>10.0	>10.0	0.35	>10.0	0.35	0.35	0.7
S7	0.7	2	2	0.35	0.35	8	>10.0	>10.0	>10.0	>10.0	8	>10.0	10	>10.0	>10.0	8	>10.0	0.7	>10.0	0.7	8	>10.0	>10.0	0.7	>10.0	>10.0
S8	0.35	0.35	0.35	0.35	0.35	4	0.35	0.35	0.35	2	0.7	0.7	0.35	>10.0	>10.0	0.35	>10.0	10	8	>10.0	>10.0	>10.0	>10.0	>10.0	>10.0	>10.0
S9	6	>10.0	4	4	>10.0	>10.0	>10.0	0.7	0.35	>10.0	>10.0	>10.0	8	>10.0	>10.0	>10.0	>10.0	>10.0	>10.0	>10.0	>10.0	>10.0	>10.0	>10.0	>10.0	>10.0
S10	0.35	2	2	10	10	0.35	0.7	0.35	10	0.35	8	0.35	0.35	0.35	0.35	0.35	2	6	10	0.35	>10.0	8	4	10	0.7	>10.0
S11	10	>10.0	>10.0	>10.0	>10.0	>10.0	>10.0	>10.0	>10.0	>10.0	4	>10.0	0.7	>10.0	>10.0	>10.0	>10.0	0.7	>10.0	>10.0	>10.0	0.35	>10.0	>10.0	>10.0	>10.0
S12	0.35	0.7	0.35	0.35	6	0.7	2	10	>10.0	0.35	2	>10.0	0.7	2	>10.0	0.35	>10.0	0.35	10	>10.0	0.7	0.35	6	0.35	4	0.7
S13	0.35	IDE	0.7	>10.0	4	>10.0	0.35	8	4	>10.0	2	>10.0	2	0.7	0.7	4	0.35	>10.0	0.7	4	0.35	0.35	0.35	0.7	>10.0	>10.0
S14	10	0.35	4	0.35	IDE	10	0.35	10	0.35	0.35	0.7	0.7	0.7	0.35	>10.0	2	0.7	0.35	0.7	4	0.7	0.7	>10.0	0.35	>10.0	>10.0
S15	6	IDE	6	6	10	0.7	>10.0	6	>10.0	>10.0	>10.0	>10.0	>10.0	>10.0	0.35	>10.0	>10.0	0.35	>10.0	>10.0	>10.0	0.7	>10.0	10	6	>10.0
S16	6	2	0.35	4	2	6	0.35	0.7	0.35	0.35	0.7	0.35	0.7	2	>10.0	4	4	2	8	4	0.7	IDE	0.7	8	2	>10.0
S17	8	>10.0	>10.0	>10.0	>10.0	0.35	>10.0	0.7	2	0.7	8	0.7	>10.0	>10.0	4	4	0.7	>10.0	0.7	>10.0	IDE	>10.0	>10.0	>10.0	10	>10.0
S18	0.7	>10.0	0.7	0.35	2	0.7	0.35	2	0.35	4	0.35	>10.0	IDE	10	>10.0	4	>10.0	0.7	>10.0	>10.0	>10.0	IDE	>10.0	0.7	0.35	6
S19	0.35	0.35	0.35	0.35	>10.0	0.35	8	IDE	4	10	0.35	0.35	>10.0	10	10	4	10	>10.0	0.35	8	>10.0	>10.0	>10.0	>10.0	4	4
S20	>10.0	>10.0	>10.0	0.35	0.35	>10.0	IDE	>10.0	>10.0	10	0.35	>10.0	10	0.7	0.7	>10.0	>10.0	>10.0	10	0.35	>10.0	>10.0	>10.0	>10.0	>10.0	>10.0
S21	0.35	IDE	0.35	0.35	0.35	0.35	IDE	0.7	2	>10.0	0.7	0.7	0.35	0.35	0.7	>10.0	0.35	10	0.7	10	0.7	>10.0	>10.0	>10.0	0.35	0.7
S22	0.35	IDE	4	0.7	0.35	2	0.35	0.35	0.35	IDE	0.35	0.35	0.7	0.35	2	0.7	>10.0	6	4	4	4	>10.0	0.35	>10.0	4	0.7
S23	0.35	0.35	0.7	4	0.7	0.7	0.7	2	IDE	4	0.7	2	6	10	IDE	0.35	4	8	0.7	6	0.7	IDE	2	0.7	8	0.35
S24	4	4	2	4	>10.0	>10.0	>10.0	>10.0	>10.0	>10.0	4	>10.0	10	8	>10.0	10	>10.0	>10.0	>10.0	8	0.35	>10.0	>10.0	>10.0	>10.0	IDE
S25	>10.0	>10.0	>10.0	>10.0	>10.0	>10.0	>10.0	>10.0	>10.0	>10.0	>10.0	>10.0	>10.0	>10.0	>10.0	>10.0	>10.0	>10.0	>10.0	>10.0	>10.0	>10.0	>10.0	IDE	>10.0	>10.0
S26	0.7	0.7	0.35	8	IDE	10	8	8	>10.0	>10.0	8	6	>10.0	0.35	10	>10.0	>10.0	>10.0	>10.0	0.7	>10.0	>10.0	IDE	>10.0	IDE	>10.0

Group 3 Subjects- Threshold Sensitivity per Location																										
Patient Identifier	Right Toe 1	Right Toe 2	Right Toe 3	Right Toe 4	Right Toe 5	Left Toe 1	Left Toe 2	Left Toe 3	Left Toe 4	Left Toe 5	Right Ball 1	Right Ball 2	Right Ball 3	Right Ball 4	Right Ball 5	Left Ball 1	Left Ball 2	Left Ball 3	Left Ball 4	Left Ball 5	Right Heel 1	Right Heel 2	Right Heel 3	Left Heel 1	Left Heel 2	Left Heel 3
NS1	0.7	0.35	>10.0	10	0.35	0.7	0.35	0.35	>10.0	0.7	2	4	0.35	0.7	>10.0	0.7	8	0.35	4	>10.0	>10.0	6	10	>10.0	10	0.35
NS2	0.35	2	0.35	0.35	0.35	0.35	0.35	0.35	0.35	0.7	0.35	0.7	0.35	0.35	4	0.35	10	0.7	0.7	2	0.35	0.7	0.7	0.35	0.35	4
NS3	10	>10.0	>10.0	>10.0	>10.0	>10.0	>10.0	>10.0	>10.0	0.35	6	>10.0	>10.0	>10.0	>10.0	10	4	>10.0	>10.0	6	2	0.35	>10.0	>10.0	>10.0	>10.0
NS4	0.35	0.35	0.35	0.35	0.35	0.35	0.35	0.35	0.35	0.35	0.35	2	0.7	0.7	0.35	0.35	0.7	0.35	0.7	0.35	0.35	0.7	0.35	0.35	0.7	0.35
NS5	0.35	8	0.35	2	0.35	2	0.7	0.35	0.35	0.7	>10.0	>10.0	10	>10.0	4	>10.0	4	0.7	10	0.7	>10.0	10	0.35	0.7	>10.0	0.7
NS6	10	2	2	0.35	0.7	0.35	0.35	0.35	0.35	8	>10.0	>10.0	>10.0	>10.0	>10.0	0.7	>10.0	>10.0	6	4	0.35	2	10	>10.0	6	>10.0
NS7	4	>10.0	8	>10.0	>10.0	>10.0	>10.0	0.7	4	0.7	0.7	>10.0	>10.0	0.7	>10.0	>10.0	>10.0	>10.0	>10.0	>10.0	>10.0	>10.0	>10.0	>10.0	>10.0	>10.0
NS8	0.35	0.35	0.35	0.7	0.35	0.35	0.35	0.35	0.35	0.7	0.35	0.35	2	6	0.35	0.35	0.35	0.35	0.7	0.35	0.35	0.7	0.35	0.35	0.7	0.7
NS9	0.35	8	0.35	0.35	2	0.35	0.35	2	2	0.35	0.7	6	0.35	6	0.7	0.7	>10.0	>10.0	6	8	0.7	10	>10.0	>10.0	>10.0	>10.0
NS10	0.7	2	0.35	0.35	0.35	4	0.7	0.35	0.35	0.7	0.7	>10.0	>10.0	>10.0	0.35	0.35	>10.0	2	>10.0	10	2	0.7	>10.0	0.7	>10.0	>10.0
NS11	0.35	0.35	2	0.35	0.35	0.35	0.35	0.35	6	0.35	0.7	2	0.35	0.35	0.7	0.7	2	2	2	6	0.35	2	0.7	0.35	4	0.7
NS12	0.35	0.35	0.35	4	0.35	>10.0	>10.0	>10.0	>10.0	>10.0	>10.0	>10.0	>10.0	0.35	0.7	>10.0	>10.0	>10.0	>10.0	>10.0	>10.0	>10.0	>10.0	>10.0	>10.0	>10.0
NS13	>10.0	2	2	>10.0	>10.0	4	2	2	6	6	>10.0	>10.0	>10.0	>10.0	>10.0	>10.0	>10.0	>10.0	>10.0	>10.0	>10.0	>10.0	>10.0	>10.0	>10.0	>10.0
NS14	0.35	IDE	0.35	0.35	0.35	0.35	0.35	0.35	0.35	0.35	0.35	0.35	0.35	0.35	0.35	0.35	0.7	0.35	0.35	0.35	0.35	0.35	4	0.35	0.35	0.35
NS15	0.35	0.35	0.7	0.7	IDE	6	0.7	0.35	0.35	0.35	IDE	0.35	0.35	0.35	0.35	>10.0	0.35	6	>10.0	0.7	10	IDE	0.7	>10.0	0.35	>10.0
NS16	0.35	IDE	0.35	0.35	0.35	0.35	IDE	0.7	0.35	0.35	0.7	0.7	0.7	0.7	0.7	0.35	>10.0	0.7	0.35	0.7	0.35	0.7	0.7	0.35	10	0.7
NS17	>10.0	0.35	>10.0	4	>10.0	4	>10.0	IDE	0.7	>10.0	>10.0	>10.0	0.7	10	>10.0	>10.0	10	>10.0	0.7	>10.0	>10.0	>10.0	IDE	>10.0	>10.0	>10.0
NS18	>10.0	0.35	>10.0	>10.0	>10.0	>10.0	>10.0	>10.0	>10.0	>10.0	>10.0	>10.0	>10.0	>10.0	>10.0	>10.0	>10.0	>10.0	IDE	>10.0	6	>10.0	>10.0	>10.0	>10.0	>10.0
NS19	0.7	0.35	0.7	0.35	0.35	0.7	>10.0	0.7	8	0.7	0.7	0.35	>10.0	0.7	>10.0	6	6	0.35	IDE	0.7	0.35	IDE	>10.0	0.35	>10.0	0.7
NS20	2	6	0.35	0.35	0.35	0.35	0.35	0.35	0.35	0.35	0.35	0.7	8	2	0.35	0.35	8	0.35	0.35	10	0.35	0.35	0.35	IDE	2	2
NS21	>10.0	0.35	0.35	0.35	0.35	0.35	2	0.35	0.7	0.35	2	0.35	0.35	0.35	2	4	0.7	0.7	2	0.7	2	>10.0	4	IDE	0.35	0.35
NS22	0.7	0.7	2	0.35	10	0.7	10	4	0.7	>10.0	0.35	0.7	0.35	0.7	0.35	>10.0	8	0.35	>10.0	0.7	0.7	IDE	>10.0	0.7	>10.0	IDE
