# Supplementary material for: Plantar threshold sensitivity assessment using an automated tool—Clinical assessment comparison between a control population without type 2 diabetes mellitus, and populations with type 2 diabetes mellitus, with and without neuropathy symptoms
Source: PLoS One. 2023 Jul 7;18(7):e0286559. doi: 10.1371/journal.pone.0286559 (PMC10328367; doi:10.1371/journal.pone.0286559)
Supplement: S1 File — (PDF) [file pone.0286559.s002.pdf]

Automated Tool Start Code-MATLAB

```
clc
clear all
close all

Hello=questdlg('Hello. Welcome to the Neuropathy Diagnostic Tool Assessment
Interface. Do not place the patient''s foot in the device yet. Please click
start to begin the test.', 'Welcome', 'Start', 'Start');
PSShcek=questdlg('Did you turn on the small power supply? If not please do so
now. Select begin test when completed.', 'Power Supply Check', 'Begin Test',
'Begin Test');
ExcelReminder=questdlg('Please make sure that Microsoft Excel is
closed.','Excel Reminder', 'Okay', 'Okay');

Mega=serial('COM3', 'BaudRate', 115200);
fopen(Mega);
pause(3)
Uno=serial('COM4', 'BaudRate', 9600);
Uno.Terminator= 'LF';
Uno.timeout=30;
fopen(Uno);
pause(3)
fprintf(Mega, '$X \n');
pause(3)
fprintf(Mega, '$H \n');
pause(3)
fprintf(Mega, 'G20 \n');
pause(3)
fprintf(Mega, 'G28.1 \n');
pause(3)
fprintf(Mega, 'G10 L20 P1 X0 Y0 Z0 \n');
pause(3)
mycam=webcam('Logitech');
mycam.Resolution = '1920x1080';
pause(10)


PatientData=inputdlg({'Patient Identifier:', 'Gender:', 'Age:', 'Time:',
'Date:', 'Operator Initials:'}, 'Documentation', [1 50]);
PatientIdentifierChar=char(PatientData(1));

answer=questdlg('Left Foot or Right Foot?', 'Foot Type', 'Left', 'Right',
'Left');

switch answer
    case 'Left'
        Type1st='LeftFoot';
        Type2nd='RightFoot';
    case 'Right'
        Type1st='RightFoot';
        Type2nd='LeftFoot';
end

Directory='D:\Documents\Graduate Research Neuropathy Project\Matlab Research
Files';
```

```
NewDirectory=sprintf('%s\\%s',Directory, PatientIdentifierChar);

if ~exist(NewDirectory, 'dir')
    mkdir(fullfile(Directory,PatientIdentifierChar))
end

PatientIdentifierCharType1st=sprintf('%s%s',PatientIdentifierChar, Type1st);
PatientIdentifierCharType2nd=sprintf('%s%s',PatientIdentifierChar, Type2nd);
SubDirectory1st=sprintf('%s\\%s', NewDirectory,
PatientIdentifierCharType1st);
SubDirectory2nd=sprintf('%s\\%s', NewDirectory,
PatientIdentifierCharType2nd);

if ~exist(SubDirectory1st, 'dir')
    mkdir(fullfile(NewDirectory, PatientIdentifierCharType1st))
end

if ~exist(SubDirectory2nd, 'dir')
    mkdir(fullfile(NewDirectory, PatientIdentifierCharType2nd))
end

filenamex1st=sprintf('%s.xlsx', PatientIdentifierCharType1st);
filenamex2nd=sprintf('%s.xlsx', PatientIdentifierCharType2nd);
filenamew=sprintf('%s.mat', PatientIdentifierChar);
filenamef1st=sprintf('%s.fig', PatientIdentifierCharType1st);
filenamef2nd=sprintf('%s.fig', PatientIdentifierCharType2nd);
tic

for H=1:2

    if H==1

        [T1, T2, T3, RS1]=NeuropathyScriptFunction2(Uno, Mega, mycam, 1);
        answer5=questdlg('Offer the Patient a break', 'Break', 'Okay',
'Okay');
        FigureReminder=questdlg('Please minimize the first window with
patient''s foot and results on it. Do not close the window.', 'Figure
Reminder', 'Okay', 'Okay');
        fprintf(Mega, '$X \n');
        pause(3)
        fprintf(Mega, '$H \n');
        pause(3)
        fprintf(Mega, 'G20 \n');
        pause(3)
        fprintf(Mega, 'G28.1 \n');
        pause(3)
        fprintf(Mega, 'G10 L20 P1 X0 Y0 Z0 \n');
        pause(3)
        answer6=questdlg('Place the Patient''s other foot in the machine',
'Insert Foot', 'Okay', 'Okay');
        answer7=questdlg('Click Okay to resume the test once the Patient''s
other foot has been placed in the machine', 'Resume Test', 'Okay', 'Okay');
    end

    if H==2

        [T4, T5, T6, RS2]=NeuropathyScriptFunction2(Uno, Mega, mycam, 2);
```

```
    end

end

endTime=toc;

fclose(Mega);
pause(3)
fclose(Uno);

SaveReminder=questdlg('Please press save to save the results. Do not close
out any windows.', 'Save Results', 'Save', 'Save');
writetable(T1, filenamex1st, 'sheet', 'sheet1')
writetable(T2, filenamex1st, 'sheet', 'sheet2')
writetable(T3, filenamex1st, 'sheet', 'sheet3')
writetable(T4, filenamex2nd, 'sheet', 'sheet1')
writetable(T5, filenamex2nd, 'sheet', 'sheet2')
writetable(T6, filenamex2nd, 'sheet', 'sheet3')

savefig(1, PatientIdentifierCharType1st)
savefig(2, PatientIdentifierCharType2nd)
save(PatientIdentifierChar)

movefile(filenamex1st, SubDirectory1st)
movefile(filenamex2nd, SubDirectory2nd)
movefile(filenamew, NewDirectory)
movefile(filenamef1st, SubDirectory1st)
movefile(filenamef2nd, SubDirectory2nd)
EndReminder=questdlg('The results have been saved and the files have been
moved into the patient''s folder. Please close all windows and close MATLAB.
Also please turn the machine off and unplug the usbs from the computer. Clean
the device before the next patient. Thank you for all your help -
Kyle.','Assessment Complete', 'Finish', 'Finish');
```

Neuropathy Script-MATLAB

```
function [T1, T2, T3, rs]=NeuropathyScriptFunction2(Uno, Mega, mycam, N)


PatientFoot1=questdlg('Please place the patient''s foot in the device now.
Make sure that the foot is strapped in place and the plantar surface is
against the acylic. Adjust the table height if necessary. Hand the patient
the remote. Press Start once the patient is ready.','Place Patient''s Foot in
Device', 'Start', 'Start');

figure(N)
img=snapshot(mycam);
imwrite(img, 'PatientNew.tiff');
I=imread('PatientNew.tiff');
imshow(I);
axis on

PatientFootImage1=questdlg('Please make the window with the picture of the
patient''s foot fullscreen. Do not interact with other windows or figures
during the test', 'Fullscreen Reminder', 'Okay', 'Okay');

%[c,r]=getpts(figure(N)); %Manual selection for grid

%c1=c(1);
%c2=c(2);

c1=103;
c2=1876;
CLength=c2-c1;
xratio=12/CLength;
cd=CLength/48;

%r1=r(1);
%r2=r(2);

r1=921;
r2=180;
RLength=r2-r1;
yratio=5/RLength;
rd=RLength/20;

hold on;

for col=c1:cd:c2
    line([col, col], [r2, r1], 'Color', 'w');
end

for row=r1:rd:r2
    line([c2, c1], [row, row], 'Color', 'w');
end

%R=r1:rd:r2;
%C=c1:cd:c2;

fprintf(Mega, 'G54 X0.26 Y1.285 \n'); %Setting New Orgin at the first hole
```

```
pause(3)
fprintf(Mega, 'G30.1 \n');
pause(3)
fprintf(Mega, 'G10 L20 P2 X0 Y0 Z0 \n');
pause(0.1)
[~, ~, ~] = NeuropathyUnoFunction2("0", Uno);
pause(0.1)


for L=1:3
    if L==1
        fprintf("Region 1-Toes \n")
        answer1=questdlg('Please select locations on the heads of each toe, 5
total. Do not double click the mouse. Backspace on the keyboard will remove
the location selected. Hit Enter on the keyboard to comfirm locations.',
'Region 1', 'Okay', 'Okay');
    end

    if L==2
        fprintf("Region 2-Ball of the Foot \n")
        answer2=questdlg('Please select locations on the ball of the foot, 5
total. Do not double click the mouse. Backspace on the keyboard will remove
the location selected. Hit Enter on the keyboard to comfirm locations.',
'Region 2', 'Okay', 'Okay');
    end

    if L==3
        fprintf("Region 3-Heel \n")
        answer3=questdlg('Please select locations on the heel, 3 total. Do
not double click the mouse. Backspace on the keyboard will remove the
location selected. Hit Enter on the keyboard to comfirm locations.', 'Region
3', 'Okay', 'Okay');
    end

[x,y]=getpts(figure(N)); %Collects data on figure
hold on

Data=[x y]; %Mouse click Data stored
DataConversion=Data-[c1 r1];
DataInch=DataConversion.*[xratio yratio];

[rowInch, ~]=size(DataInch); %Determines how many points there are
NumberRows=rowInch; %Counts rows

accuracy=0.25; %rounded to nearest 0.25 inch
DataRounded=round(DataInch/accuracy)*accuracy;
DataRoundedLimit=max(DataRounded, 0); %Converts negative numbers to zero

XData=DataRoundedLimit(: ,1); %X axis data to less than or equal to 12
XData(XData>12)=12;

YData=DataRoundedLimit(: ,2); %Y axis data to less than or equal to 5
YData(YData>5)=5;

DataFinal=[XData YData];
DataFinalPixels=DataFinal./[xratio yratio];
```

```
DataPixels=DataFinalPixels+[c1 r1];
DataPixelsX=DataPixels(: ,1);
DataPixelsY=DataPixels(: ,2);
plot(DataPixelsX,DataPixelsY, 'c*');
hold on

GcodeCell=cell(1, NumberRows);
for i=1:NumberRows
    DataPoint=DataFinal(i, :); %Selects a row of data for indexed value or
rounded set
    DataPointX=DataPoint(2); %X coorrdinate for indexed value
    DataPointY=DataPoint(1); %Y coordinate for indexed value
    Gcode=sprintf('G55 X%0.2f Y%0.2f', DataPointX, DataPointY); %String
printf of Gcode
    GcodeCell{i}=Gcode;  %Stores all indexed Gcode in a Cell
end

    if L==1
        R1Gcode=GcodeCell;
        R1DataPixels=DataPixels;
        R1DataPixelsX=DataPixelsX;
        R1DataPixelsY=DataPixelsY;
        Length1=NumberRows;
    end

    if L==2
        R2Gcode=GcodeCell;
        R2DataPixels=DataPixels;
        R2DataPixelsX=DataPixelsX;
        R2DataPixelsY=DataPixelsY;
        Length2=NumberRows;
    end

    if L==3
        R3Gcode=GcodeCell;
        R3DataPixels=DataPixels;
        R3DataPixelsX=DataPixelsX;
        R3DataPixelsY=DataPixelsY;
        Length3=NumberRows;
    end
end

rng shuffle
rs=rng;

TestingOrder=randi(4);
if TestingOrder==1
    %1-2-3

    T1Gcode=R1Gcode;
    T1DataPixels=R1DataPixels;
    T1DataPixelsX=R1DataPixelsX;
    T1DataPixelsY=R1DataPixelsY;
    T1Length=Length1;

    T2Gcode=R2Gcode;
    T2DataPixels=R2DataPixels;
```

```
    T2DataPixelsX=R2DataPixelsX;
    T2DataPixelsY=R2DataPixelsY;
    T2Length=Length2;

    T3Gcode=R3Gcode;
    T3DataPixels=R3DataPixels;
    T3DataPixelsX=R3DataPixelsX;
    T3DataPixelsY=R3DataPixelsY;
    T3Length=Length3;

elseif TestingOrder==2
    %2-1-3

    T1Gcode=R2Gcode;
    T1DataPixels=R2DataPixels;
    T1DataPixelsX=R2DataPixelsX;
    T1DataPixelsY=R2DataPixelsY;
    T1Length=Length2;

    T2Gcode=R1Gcode;
    T2DataPixels=R1DataPixels;
    T2DataPixelsX=R1DataPixelsX;
    T2DataPixelsY=R1DataPixelsY;
    T2Length=Length1;

    T3Gcode=R3Gcode;
    T3DataPixels=R3DataPixels;
    T3DataPixelsX=R3DataPixelsX;
    T3DataPixelsY=R3DataPixelsY;
    T3Length=Length3;

elseif TestingOrder==3
    %3-1-2

    T1Gcode=R3Gcode;
    T1DataPixels=R3DataPixels;
    T1DataPixelsX=R3DataPixelsX;
    T1DataPixelsY=R3DataPixelsY;
    T1Length=Length3;

    T2Gcode=R1Gcode;
    T2DataPixels=R1DataPixels;
    T2DataPixelsX=R1DataPixelsX;
    T2DataPixelsY=R1DataPixelsY;
    T2Length=Length1;

    T3Gcode=R2Gcode;
    T3DataPixels=R2DataPixels;
    T3DataPixelsX=R2DataPixelsX;
    T3DataPixelsY=R2DataPixelsY;
    T3Length=Length2;

else
    %3-2-1

    T1Gcode=R3Gcode;
    T1DataPixels=R3DataPixels;
```

```
    T1DataPixelsX=R3DataPixelsX;
    T1DataPixelsY=R3DataPixelsY;
    T1Length=Length3;

    T2Gcode=R2Gcode;
    T2DataPixels=R2DataPixels;
    T2DataPixelsX=R2DataPixelsX;
    T2DataPixelsY=R2DataPixelsY;
    T2Length=Length2;

    T3Gcode=R1Gcode;
    T3DataPixels=R1DataPixels;
    T3DataPixelsX=R1DataPixelsX;
    T3DataPixelsY=R1DataPixelsY;
    T3Length=Length1;
end

for K=1:3

    if K==1
        [T1] = NeuropathyFunction4(T1Length, T1Gcode, T1DataPixelsX,
T1DataPixelsY, Mega, Uno);
    end

    if K==2
        [T2] = NeuropathyFunction4(T2Length, T2Gcode, T2DataPixelsX,
T2DataPixelsY, Mega, Uno);
    end

    if K==3
        [T3] = NeuropathyFunction4(T3Length, T3Gcode, T3DataPixelsX,
T3DataPixelsY, Mega, Uno);
    end

end

fprintf(Mega, 'G30 \n');
pause(3)
answer4=questdlg('Please remove the Patient''s foot and click okay when
done', 'Remove Foot', 'Okay', 'Okay');
```

Neuropathy Function-MATLAB

```
function [T] = NeuropathyFunction4(TLength, TGcode, TDataPixelsX,
TDataPixelsY, Mega, Uno)

TotalNumberofTrialVector=1:TLength; %Creates row vector of total number of
points selected
VectorTranspose=TotalNumberofTrialVector'; %Creates cloumn vector of total
number of points selected
RandomizedVector=TotalNumberofTrialVector(randperm(length(TotalNumberofTrialV
ector))); %Randomizes the vector, used to randomize the test locations
RandomizedVectorTranspose=RandomizedVector'; %Takes random vector and
transposes it
Y=RandomizedVector;

GcodeCelltoString=string(TGcode); %Converts cell array Gcode to strings
GcodeCelltoStringTranspose=GcodeCelltoString'; %Transposes a Gcode string
array
Z=GcodeCelltoString;

FalsePositiveRandomizationVector=randi([1 10],1,TLength); %Flase Positive
Randmization
FalsePositiveRandomizationVectorTranspose=FalsePositiveRandomizationVector';
%Flase Positive Randomization Transpose
W=FalsePositiveRandomizationVector;
X=TLength;

TestLocationCellArray=cell(1, X);
dataout0=zeros(1, X);
dataout1=zeros(1, X);
dataout2=zeros(1, X);
dataout3=zeros(1, X);
dataout4=zeros(1, X);
dataout5=zeros(1, X);
dataout6=zeros(1, X);
dataout7=zeros(1, X);
ResponseIndex0=zeros(1,X);
ResponseIndex1=zeros(1,X);
ResponseIndex2=zeros(1,X);
ResponseIndex3=zeros(1,X);
ResponseIndex4=zeros(1,X);
ResponseIndex5=zeros(1,X);
ResponseIndex6=zeros(1,X);
ResponseIndex7=zeros(1,X);
PercentError0=zeros(1,X);
PercentError1=zeros(1,X);
PercentError2=zeros(1,X);
PercentError3=zeros(1,X);
PercentError4=zeros(1,X);
PercentError5=zeros(1,X);
PercentError6=zeros(1,X);
PercentError7=zeros(1,X);
FalsePositiveCheckIndex=zeros(1,X);
RS=cell(1,X);

for i=1:X
```

```
    TestLocation=Z(Y(i)); %Selects a random point to test at
    fprintf(Mega, '%s  \n',TestLocation); %Sends Gcode (X and Y) to the
arduino over serial usb connection
    TestLocationCellArray{i}=TestLocation; %Stores location data
    fo=0;

        while fo==0 %feedback loop
            flushinput(Mega);
            pause(1)
            fprintf(Mega, '? \n');
            C=fscanf(Mega);
            CC=strtok(C, '|');
            CCC=convertCharsToStrings(CC);

                if CCC=="<Idle"
                    fo=1;
                end
        end

    for j=1

        if W(i)==10
            %false positive code
            FalsePositiveCheckRandomizationVector=randi(2);
            FalsePositiveCheckIndex(i)=FalsePositiveCheckRandomizationVector;

            if FalsePositiveCheckRandomizationVector==2
                fprintf('False Positive Test at End \n');
                fprintf('Z axis move \n');
                pause(0.1);
                F="0.35";
                [ResponseIndex1(i), dataout1(i), PercentError1(i)] =
NeuropathyUnoFunction2(F, Uno);

                if ResponseIndex1(i)==1
                    ResponseIndex2(i)=101;
                    ResponseIndex3(i)=101;
                    ResponseIndex4(i)=101;
                    ResponseIndex5(i)=101;
                    ResponseIndex6(i)=101;
                    ResponseIndex7(i)=101;
                    dataout2(i)=0;
                    dataout3(i)=0;
                    dataout4(i)=0;
                    dataout5(i)=0;
                    dataout6(i)=0;
                    dataout7(i)=0;
                    PercentError2(i)=0;
                    PercentError3(i)=0;
                    PercentError4(i)=0;
                    PercentError5(i)=0;
                    PercentError6(i)=0;
                    PercentError7(i)=0;
                    RS{i}="0.350 grams";
                end

                if ResponseIndex1(i)==0
```

```
                    fprintf('Z axis move \n'); %Z axis moves
                    pause(0.1);
                    F="10";
                    [ResponseIndex2(i), dataout2(i), PercentError2(i)] =
NeuropathyUnoFunction2(F, Uno);
                end

                if ResponseIndex2(i)==1
                    fprintf('Z axis move \n'); %Z axis moves
                    pause(0.1);
                    F="4";
                    [ResponseIndex3(i), dataout3(i), PercentError3(i)] =
NeuropathyUnoFunction2(F, Uno);
                end

                if ResponseIndex2(i)==0
                    ResponseIndex3(i)=101;
                    ResponseIndex4(i)=101;
                    ResponseIndex5(i)=101;
                    ResponseIndex6(i)=101;
                    ResponseIndex7(i)=101;
                    dataout3(i)=0;
                    dataout4(i)=0;
                    dataout5(i)=0;
                    dataout6(i)=0;
                    dataout7(i)=0;
                    PercentError3(i)=0;
                    PercentError4(i)=0;
                    PercentError5(i)=0;
                    PercentError6(i)=0;
                    PercentError7(i)=0;
                    RS{i}="Greater than 10.0 grams";
                end

                if ResponseIndex3(i)==1
                    fprintf('Z axis move \n'); %Z axis moves
                    pause(0.1);
                    F="0.7";
                    [ResponseIndex4(i), dataout4(i), PercentError4(i)] =
NeuropathyUnoFunction2(F, Uno);

                    if ResponseIndex4(i)==1
                        ResponseIndex5(i)=101;
                        ResponseIndex6(i)=101;
                        ResponseIndex7(i)=101;
                        dataout5(i)=0;
                        dataout6(i)=0;
                        dataout7(i)=0;
                        PercentError5(i)=0;
                        PercentError6(i)=0;
                        PercentError7(i)=0;
                        RS{i}="0.700 grams";
                    end

                    if ResponseIndex4(i)==0
                        fprintf('Z axis move \n'); %Z axis moves
                        pause(0.1);
```

```
                        F="2";
                        [ResponseIndex6(i), dataout6(i), PercentError6(i)] =
NeuropathyUnoFunction2(F, Uno);

                        ResponseIndex5(i)=101;
                        ResponseIndex7(i)=101;
                        dataout5(i)=0;
                        dataout7(i)=0;
                        PercentError5(i)=0;
                        PercentError7(i)=0;
                    end

                    if ResponseIndex6(i)==1
                       RS{i}="2.00 grams";
                    end

                    if ResponseIndex6(i)==0
                       RS{i}="4.00 grams";
                    end

                end

                if ResponseIndex3(i)==0
                    fprintf('Z axis move \n'); %Z axis moves
                    pause(0.1);
                    F="8";
                    [ResponseIndex5(i), dataout5(i), PercentError5(i)] =
NeuropathyUnoFunction2(F, Uno);

                    if ResponseIndex5(i)==1
                        fprintf('Z axis move \n'); %Z axis moves
                        pause(0.1);
                        F="6";
                        [ResponseIndex7(i), dataout7(i), PercentError7(i)] =
NeuropathyUnoFunction2(F, Uno);

                        ResponseIndex4(i)=101;
                        ResponseIndex6(i)=101;
                        dataout4(i)=0;
                        dataout6(i)=0;
                        PercentError4(i)=0;
                        PercentError6(i)=0;
                    end

                    if ResponseIndex7(i)==1
                        RS{i}="6.00 grams";
                    end

                    if ResponseIndex7(i)==0
                        RS{i}="8.00 grams";
                    end

                    if ResponseIndex5(i)==0
                        ResponseIndex4(i)=101;
                        ResponseIndex6(i)=101;
                        ResponseIndex7(i)=101;
                        dataout4(i)=0;
```

```
                dataout6(i)=0;
                dataout7(i)=0;
                PercentError4(i)=0;
                PercentError6(i)=0;
                PercentError7(i)=0;
                RS{i}="10.0 grams";
            end

        end

        fprintf('False Positive Z \n');
        pause(0.1);
        F="0";
        [ResponseIndex0(i), dataout0(i), PercentError0(i)] =
NeuropathyUnoFunction2(F, Uno);

    else
        fprintf('False Positive at Beginning \n');
        fprintf('False Positive Z \n');
        pause(0.1);
        F="0";
        [ResponseIndex0(i), dataout0(i), PercentError0(i)] =
NeuropathyUnoFunction2(F, Uno);

        fprintf('Z axis move \n');
        pause(0.1);
        F="0.35";
        [ResponseIndex1(i), dataout1(i), PercentError1(i)] =
NeuropathyUnoFunction2(F, Uno);

        if ResponseIndex1(i)==1
            ResponseIndex2(i)=101;
            ResponseIndex3(i)=101;
            ResponseIndex4(i)=101;
            ResponseIndex5(i)=101;
            ResponseIndex6(i)=101;
            ResponseIndex7(i)=101;
            dataout2(i)=0;
            dataout3(i)=0;
            dataout4(i)=0;
            dataout5(i)=0;
            dataout6(i)=0;
            dataout7(i)=0;
            PercentError2(i)=0;
            PercentError3(i)=0;
            PercentError4(i)=0;
            PercentError5(i)=0;
            PercentError6(i)=0;
            PercentError7(i)=0;
            RS{i}="0.350 grams";
        end

        if ResponseIndex1(i)==0
            fprintf('Z axis move \n'); %Z axis moves
            pause(0.1);
            F="10";
```

```
                    [ResponseIndex2(i), dataout2(i), PercentError2(i)] =
NeuropathyUnoFunction2(F, Uno);
                end

                if ResponseIndex2(i)==1
                    fprintf('Z axis move \n'); %Z axis moves
                    pause(0.1);
                    F="4";
                    [ResponseIndex3(i), dataout3(i), PercentError3(i)] =
NeuropathyUnoFunction2(F, Uno);
                end

                if ResponseIndex2(i)==0
                    ResponseIndex3(i)=101;
                    ResponseIndex4(i)=101;
                    ResponseIndex5(i)=101;
                    ResponseIndex6(i)=101;
                    ResponseIndex7(i)=101;
                    dataout3(i)=0;
                    dataout4(i)=0;
                    dataout5(i)=0;
                    dataout6(i)=0;
                    dataout7(i)=0;
                    PercentError3(i)=0;
                    PercentError4(i)=0;
                    PercentError5(i)=0;
                    PercentError6(i)=0;
                    PercentError7(i)=0;
                    RS{i}="Greater than 10.0 grams";
                end

                if ResponseIndex3(i)==1
                    fprintf('Z axis move \n'); %Z axis moves
                    pause(0.1);
                    F="0.7";
                    [ResponseIndex4(i), dataout4(i), PercentError4(i)] =
NeuropathyUnoFunction2(F, Uno);

                    if ResponseIndex4(i)==1
                        ResponseIndex5(i)=101;
                        ResponseIndex6(i)=101;
                        ResponseIndex7(i)=101;
                        dataout5(i)=0;
                        dataout6(i)=0;
                        dataout7(i)=0;
                        PercentError5(i)=0;
                        PercentError6(i)=0;
                        PercentError7(i)=0;
                        RS{i}="0.700 grams";
                    end

                    if ResponseIndex4(i)==0
                        fprintf('Z axis move \n'); %Z axis moves
                        pause(0.1);
                        F="2";
                        [ResponseIndex6(i), dataout6(i), PercentError6(i)] =
NeuropathyUnoFunction2(F, Uno);
```

```
                ResponseIndex5(i)=101;
                ResponseIndex7(i)=101;
                dataout5(i)=0;
                dataout7(i)=0;
                PercentError5(i)=0;
                PercentError7(i)=0;
            end

            if ResponseIndex6(i)==1
               RS{i}="2.00 grams";
            end

            if ResponseIndex6(i)==0
               RS{i}="4.00 grams";
            end

        end

        if ResponseIndex3(i)==0
            fprintf('Z axis move \n'); %Z axis moves
            pause(0.1);
            F="8";
            [ResponseIndex5(i), dataout5(i), PercentError5(i)] =
NeuropathyUnoFunction2(F, Uno);

            if ResponseIndex5(i)==1
                fprintf('Z axis move \n'); %Z axis moves
                pause(0.1);
                F="6";
                [ResponseIndex7(i), dataout7(i), PercentError7(i)] =
NeuropathyUnoFunction2(F, Uno);

                ResponseIndex4(i)=101;
                ResponseIndex6(i)=101;
                dataout4(i)=0;
                dataout6(i)=0;
                PercentError4(i)=0;
                PercentError6(i)=0;
            end

            if ResponseIndex7(i)==1
                RS{i}="6.00 grams";
            end

            if ResponseIndex7(i)==0
                RS{i}="8.00 grams";
            end

            if ResponseIndex5(i)==0
                ResponseIndex4(i)=101;
                ResponseIndex6(i)=101;
                ResponseIndex7(i)=101;
                dataout4(i)=0;
                dataout6(i)=0;
                dataout7(i)=0;
                PercentError4(i)=0;
```

```
                        PercentError6(i)=0;
                        PercentError7(i)=0;
                        RS{i}="10.0 grams";
                    end

                end

            end

        else
            fprintf('No False Positive \n');
            fprintf('Z axis move \n'); %Z axis moves
            pause(0.1);
            F="0.35";
            [ResponseIndex1(i), dataout1(i), PercentError1(i)] =
NeuropathyUnoFunction2(F, Uno);
            FalsePositiveCheckIndex(i)=0;

            if ResponseIndex1(i)==1
                    ResponseIndex0(i)=101;
                    ResponseIndex2(i)=101;
                    ResponseIndex3(i)=101;
                    ResponseIndex4(i)=101;
                    ResponseIndex5(i)=101;
                    ResponseIndex6(i)=101;
                    ResponseIndex7(i)=101;
                    dataout0(i)=0;
                    dataout2(i)=0;
                    dataout3(i)=0;
                    dataout4(i)=0;
                    dataout5(i)=0;
                    dataout6(i)=0;
                    dataout7(i)=0;
                    PercentError0(i)=0;
                    PercentError2(i)=0;
                    PercentError3(i)=0;
                    PercentError4(i)=0;
                    PercentError5(i)=0;
                    PercentError6(i)=0;
                    PercentError7(i)=0;
                    RS{i}="0.350 grams";
            end

            if ResponseIndex1(i)==0
                fprintf('Z axis move \n'); %Z axis moves
                pause(0.1);
                F="10";
                [ResponseIndex2(i), dataout2(i), PercentError2(i)] =
NeuropathyUnoFunction2(F, Uno);
            end

            if ResponseIndex2(i)==1
                fprintf('Z axis move \n'); %Z axis moves
                pause(0.1);
                F="4";
                [ResponseIndex3(i), dataout3(i), PercentError3(i)] =
NeuropathyUnoFunction2(F, Uno);
```

```
            end

            if ResponseIndex2(i)==0
                    ResponseIndex0(i)=101;
                    ResponseIndex3(i)=101;
                    ResponseIndex4(i)=101;
                    ResponseIndex5(i)=101;
                    ResponseIndex6(i)=101;
                    ResponseIndex7(i)=101;
                    dataout0(i)=0;
                    dataout3(i)=0;
                    dataout4(i)=0;
                    dataout5(i)=0;
                    dataout6(i)=0;
                    dataout7(i)=0;
                    PercentError0(i)=0;
                    PercentError3(i)=0;
                    PercentError4(i)=0;
                    PercentError5(i)=0;
                    PercentError6(i)=0;
                    PercentError7(i)=0;
                    RS{i}="Greater than 10.0 grams";
            end

            if ResponseIndex3(i)==1
                fprintf('Z axis move \n'); %Z axis moves
                pause(0.1);
                F="0.7";
                [ResponseIndex4(i), dataout4(i), PercentError4(i)] =
NeuropathyUnoFunction2(F, Uno);

                if ResponseIndex4(i)==1
                        ResponseIndex0(i)=101;
                        ResponseIndex5(i)=101;
                        ResponseIndex6(i)=101;
                        ResponseIndex7(i)=101;
                        dataout0(i)=0;
                        dataout5(i)=0;
                        dataout6(i)=0;
                        dataout7(i)=0;
                        PercentError0(i)=0;
                        PercentError5(i)=0;
                        PercentError6(i)=0;
                        PercentError7(i)=0;
                        RS{i}="0.700 grams";
                end

                if ResponseIndex4(i)==0
                    fprintf('Z axis move \n'); %Z axis moves
                    pause(0.1);
                    F="2";
                    [ResponseIndex6(i), dataout6(i), PercentError6(i)] =
NeuropathyUnoFunction2(F, Uno);

                    ResponseIndex0(i)=101;
                    ResponseIndex5(i)=101;
                    ResponseIndex7(i)=101;
```

```
                dataout0(i)=0;
                dataout5(i)=0;
                dataout7(i)=0;
                PercentError0(i)=0;
                PercentError5(i)=0;
                PercentError7(i)=0;
            end

            if ResponseIndex6(i)==1
               RS{i}="2.00 grams";
            end

            if ResponseIndex6(i)==0
               RS{i}="4.00 grams";
            end

        end

        if ResponseIndex3(i)==0
            fprintf('Z axis move \n'); %Z axis moves
            pause(0.1);
            F="8";
            [ResponseIndex5(i), dataout5(i), PercentError5(i)] =
NeuropathyUnoFunction2(F, Uno);

            if ResponseIndex5(i)==1
                fprintf('Z axis move \n'); %Z axis moves
                pause(0.1);
                F="6";
                [ResponseIndex7(i), dataout7(i), PercentError7(i)] =
NeuropathyUnoFunction2(F, Uno);

                ResponseIndex0(i)=101;
                ResponseIndex4(i)=101;
                ResponseIndex6(i)=101;
                dataout0(i)=0;
                dataout4(i)=0;
                dataout6(i)=0;
                PercentError0(i)=0;
                PercentError4(i)=0;
                PercentError6(i)=0;
            end

            if ResponseIndex7(i)==1
                RS{i}="6.00 grams";
            end

            if ResponseIndex7(i)==0
                RS{i}="8.00 grams";
            end

            if ResponseIndex5(i)==0
                ResponseIndex0(i)=101;
                ResponseIndex4(i)=101;
                ResponseIndex6(i)=101;
                ResponseIndex7(i)=101;
                dataout0(i)=0;
```

```
                    dataout4(i)=0;
                    dataout6(i)=0;
                    dataout7(i)=0;
                    PercentError0(i)=0;
                    PercentError4(i)=0;
                    PercentError6(i)=0;
                    PercentError7(i)=0;
                    RS{i}="10.0 grams";
                end

            end

        end
    end
end

TestLocationCelltoString=string(TestLocationCellArray);
TestLocationCelltoStringTranspose=TestLocationCelltoString';
ResponseIndexRecordI0=ResponseIndex0';
ResponseIndexRecordI1=ResponseIndex1';
ResponseIndexRecordI2=ResponseIndex2';
ResponseIndexRecordI3=ResponseIndex3';
ResponseIndexRecordI4=ResponseIndex4';
ResponseIndexRecordI5=ResponseIndex5';
ResponseIndexRecordI6=ResponseIndex6';
ResponseIndexRecordI7=ResponseIndex7';

FalsePositiveCheckIndexTranspose=FalsePositiveCheckIndex';
ForceIndexRecordD0=dataout0';
ForceIndexRecordD1=dataout1';
ForceIndexRecordD2=dataout2';
ForceIndexRecordD3=dataout3';
ForceIndexRecordD4=dataout4';
ForceIndexRecordD5=dataout5';
ForceIndexRecordD6=dataout6';
ForceIndexRecordD7=dataout7';
PercentErrorC0=PercentError0';
PercentErrorC1=PercentError1';
PercentErrorC2=PercentError2';
PercentErrorC3=PercentError3';
PercentErrorC4=PercentError4';
PercentErrorC5=PercentError5';
PercentErrorC6=PercentError6';
PercentErrorC7=PercentError7';

NumberofTrials=VectorTranspose;
LocationSelected=GcodeCelltoStringTranspose;
RandomizedOrder=RandomizedVectorTranspose;
RandomizedLocations=TestLocationCelltoStringTranspose;

FalsePositiveCheck=FalsePositiveRandomizationVectorTranspose;
FalsePositiveOrderofOccurance=FalsePositiveCheckIndexTranspose;

RandomizedPixelsX=TDataPixelsX(RandomizedVector);
RandomizedPixelsY=TDataPixelsY(RandomizedVector);

RR=RS';
```

```
RRS=string(RR);
RRC=convertStringsToChars(RRS);
colors=[0 1 0; 0 1 1; 0 0 1; 1 1 0; 0.9290 0.6940 0.1250; 1 0 1; 1 0 0; 0 0
0];
XC=[ 0 0 0 0 0 0 0 0]';
YC=XC;

GC=[ "0.350 grams" "0.700 grams" "2.00 grams" "4.00 grams" "6.00 grams" "8.00
grams" "10.0 grams" "Greater than 10.0 grams"]';
GF=[GC; RRC];
XF=[XC; RandomizedPixelsX];
YF=[YC; RandomizedPixelsY];
gscatter(XF, YF, GF, colors, '', 30, 'on', '', '');
legend('Location','northeastoutside');
hold on

T=table(NumberofTrials, LocationSelected, RandomizedOrder,
RandomizedLocations, FalsePositiveCheck, ForceIndexRecordD0, PercentErrorC0,
ResponseIndexRecordI0, FalsePositiveOrderofOccurance, ForceIndexRecordD1,
PercentErrorC1, ResponseIndexRecordI1, ForceIndexRecordD2, PercentErrorC2,
ResponseIndexRecordI2, ForceIndexRecordD3, PercentErrorC3,
ResponseIndexRecordI3, ForceIndexRecordD4, PercentErrorC4,
ResponseIndexRecordI4, ForceIndexRecordD5, PercentErrorC5,
ResponseIndexRecordI5, ForceIndexRecordD6, PercentErrorC6,
ResponseIndexRecordI6, ForceIndexRecordD7, PercentErrorC7,
ResponseIndexRecordI7);

End
```

Neuropathy Uno Function-MATLAB

```
function [ResponseIndex, dataoutnum, PercentError] =
NeuropathyUnoFunction2(F, Uno)


flushinput(Uno);

%Uno=serial('COM6', 'BaudRate', 9600);
%fopen(Uno);
%pause(3)

fprintf(Uno,'%s\n', F);
foo=0;

while foo==0

    if Uno.BytesAvailable>=0
        dataoutLC=fscanf(Uno,'%s\n');
        dataoutH=fscanf(Uno,'%s\n');
        foo=1;
    end
end

if dataoutH=="y"
    ResponseIndex=1;
else
    ResponseIndex=0;
end

dataoutstr=convertCharsToStrings(dataoutLC);
dataoutnum=str2double(dataoutstr);
Fnum=str2double(F);

if Fnum==0
    PercentError=0;
else
PercentError=abs((Fnum-dataoutnum)/Fnum)*100;
end

%pause(0.25)
%fclose(Uno);
%pause(0.25)
%flushoutput(Uno);

end
```

Arduino Uno Function

```
#include "Arduino.h"
#include "HX711N.h"
#include "AccelStepper.h"
#include <avr/wdt.h>

HX711N scale(3, 2);  // (DOUT, CLK)

double measuredload;
char response;
bool stop_motor = false;
int calibration_factor = 13280;
int trim_in_pin = A0;
int trim_value = 0;

// Motor steps per revolution. Most steppers are 200 steps or 1.8
degrees/step
#define MOTOR_STEPS 200

// Microstepping mode. If you hardwired it to save pins, set to the same
value here.
#define MICROSTEPS 1

#define DIR 6
#define STEP 7

bool foo = 1;
bool foot = 0;
int foob = 1;
bool fee=0;
int fum=0;

AccelStepper stepper(AccelStepper::DRIVER, STEP, DIR); // Defaults to
AccelStepper::FULL4WIRE (4 pins) on 2, 3, 4, 5

float inputload;
int startt;
int endt;
int n;

void setup() {
  pinMode(8, INPUT_PULLUP);
  pinMode(9, OUTPUT);
  stepper.setMaxSpeed(1000);
  stepper.setSpeed(-100);

  Serial.begin(9600);
  //  Serial.println("HX711 calibration sketch");
  //  Serial.println("Remove all weight from scale");
  //  Serial.println("After readings begin, place known weight on scale");

  //  scale.set_scale();
  scale.tare(10); // Reset the scale to 0; Reads value 10 times, calculates
average and uses it as offset.
```

```
  //  long zero_factor = scale.read_average(); //Get a baseline reading
  //  Serial.print("Zero factor: "); //This can be used to remove the need to
tare the scale. Useful in permanent scale projects.
  //  Serial.println(zero_factor);
  //
  scale.set_scale(calibration_factor); //Adjust to this calibration factor
  stepper.setCurrentPosition(0);
}

void loop() {

  if(Serial.available()>0 && foot == 0 && foo == 1)
  {
    inputload = Serial.parseFloat();
    foot = 1;
    foo = 0;
  }
  // NOTE: LOOP HAS TO RUN QUICKLY FOR STEPPER TO MOVE SMOOTHLY. DO NOT ADD
DELAYS
  while (foot == 1){
    stepper.setSpeed(-100);
    stepper.setAcceleration(250);
    stepper.move(-300);
    stepper.runToPosition();
    foot = 0;
    delay(250);
    }

  if (scale.is_ready() && foo == 0){
    measuredload = scale.get_units_direct();

    if (measuredload < .25*inputload) {
      stop_motor = false;
      stepper.setSpeed(-20);
    }
    else if (measuredload < .5*inputload) {
      stop_motor = false;
      stepper.setSpeed(-15);
    }
    else if (measuredload < .75*inputload) {
      stop_motor = false;
      stepper.setSpeed(-10);
    }
    else if (measuredload < .9*inputload) {
      stop_motor = false;
      stepper.setSpeed(-7);
    }
    else if (measuredload < 1*inputload) {
      stop_motor = false;
      stepper.setSpeed(-5);
    }
    else {
      stop_motor = true;
      foo=1;
    }

  }
```

```
if (stop_motor) {
  Serial.println(measuredload);
  stepper.moveTo(0);
  stepper.setSpeed(200);
  fee=0;
  while (fee==0){
    stepper.runToPosition();

    fee=1;

  }

  foo=1;
  foob = 1;
  fum=0;
      startt = millis();
      endt = startt;
          while ((endt - startt) <=5000 && fum==0){
              if (foob==1){

              if (n%2==0){
              digitalWrite(9, HIGH);
              delay(250);
              }

              if (n%2!=0){
              digitalWrite(9, LOW);
              delay(250);
              }

                    if (digitalRead(8) == LOW){
                      response = 'y';
                      Serial.println(response);
                      //Serial.println("felt");
                      digitalWrite(9, HIGH);
                      foob = 2;
                      fum=1;
                    }
              endt = millis();
              n=n+1;
        }
        }
          if (digitalRead(8) == HIGH && foo == 1){
          response = 'n';
          Serial.println(response);
          foob = 0;
          digitalWrite(9, LOW);
          }

          while (foob==0 || foob==2){
          //Serial.end();
          foo=0;
          reboot();

          }
```

```
            //Serial.end();
  }

  else{
    stepper.runSpeed();
  }

}

void reboot(){
  wdt_disable();
  wdt_enable(WDTO_15MS);
  while (1) {}
}
```

Arduino Mega-GRBL Settings

Grbl 1.1g ['$' for help]
[MSG:'$H'|'$X' to unlock]
$0=10
$1=255
$2=0
$3=0
$4=0
$5=0
$6=0
$10=3
$11=0.010
$12=0.002
$13=1
$20=0
$21=1
$22=1
$23=3
$24=25.000
$25=500.000
$26=25
$27=1.000
$30=1000
$31=0
$32=0
$100=500.000
$101=500.000
$102=100.000
$110=1000.000
$111=1000.000
$112=100.000
$120=100.000
$121=100.000
$122=1.000
$130=200.000
$131=200.000
$132=200.000
